# A scoping study of low-wage workers’ retirement in the U.S. after the Great Recession

**DOI:** 10.1093/geroni/igab046.2286

**Published:** 2021-12-17

**Authors:** Hyesu Yeo

**Affiliations:** University of Georgia, Athens, Georgia, United States

## Abstract

Background Retirement comes with high risks for low-wage workers because of their cumulative disadvantages, and the Great Recession aggravated this population's working lives. However, there has been a lack of research about this vulnerable population's retirement. The purpose of this scoping study is to offer a comprehensive understanding of low-wage workers' retirement after the Great Recession. Methods Based on the rigorous method of scoping review by Arskey and O'Mally, the researcher systematically searched, selected, and synthesized literature. The articles were collected from eight databases and were published in January 2008 - February 2019. The search terms included terms related to retirement and low-wage. After systemically reviewing titles, abstracts, and full-texts from 5,268 articles, the final chart contains sample characteristics, definitions of low-wage workers, policy/programs, etc., from 23 peer-reviewed empirical studies. Results The results indicated that: 1) Most of the retirement studies covered middle-wage class workers, excluding low-wage workers because of their lack of retirement affordability; 2) There was no common definition of low-wage workers among scholars; 3) 11 studies assumed Social Security is the only retirement income for low-wage workers, and 12 studies investigated how to improve the workers' participation in other retirement programs; 4) Most studies were economic-centered; 5) Low-wage workers had different socioeconomic and labor market characteristics. Conclusion and Implications First, a consensus on the definition of low-wage is required to improve policies and programs associated with this population's retirement. Second, the life-course perspective approach from various disciplines is necessary to improve low-wage workers' retirement, considering diverse backgrounds.

